# Multiple Cervical Arteries Dissection Associated With Fibromuscular Dysplasia: A Case Report

**DOI:** 10.1002/ccr3.70680

**Published:** 2025-07-27

**Authors:** Julia Goginski, Renato Fedatto Beraldo, Ricardo Nascimento Brito, Gianluca Scalia, Giovanni Federico Nicoletti, Lohana Pompelli Scapatici, Bruno Liebl, Zeferino Demartini Junior, Bipin Chaurasia

**Affiliations:** ^1^ Department of Neurosurgery Hospital Universitário Cajuru – PUCPR Curitiba PR Brazil; ^2^ Department of Neurosurgery Hospital de Clinicas – UFPR Curitiba PR Brazil; ^3^ Neurosurgery Unit, Department of Head and Neck Surgery Garibaldi Hospital Catania Italy; ^4^ Department of Neurosurgery College of Medical Sciences Bharatpur Nepal

**Keywords:** case reports, dissection artery, fibromuscular dysplasia, internal carotid artery dissection, neurosurgery, neurosurgical procedures, vertebral artery dissection

## Abstract

Spontaneous cervical artery dissection is a condition often associated with connective tissue diseases, such as fibromuscular dysplasia (FMD), a complex angiopathy primarily affecting medium‐sized arteries. While single‐vessel dissection is more common, simultaneous involvement of three cervical arteries is rare. We present the case of a 30‐year‐old female patient who suffered from bilateral cerebellar infarction, subarachnoid hemorrhage, and hydrocephalus, necessitating external ventricular drainage and posterior fossa decompressive craniectomy. Multimodal imaging, including computed tomography angiography (CTA), magnetic resonance angiography (MRA), and digital subtraction angiography (DSA), confirmed the presence of FMD with dissections involving the right internal carotid artery and both vertebral arteries. Following a two‐month course of antiplatelet therapy, the patient experienced minimal residual symptoms, enabling independent daily activities. Given that FMD is an underrecognized cause of multiple cervical artery dissections, clinicians should maintain a high index of suspicion when encountering such cases. This case highlights the importance of thorough investigation and management in similar clinical presentations.

**Summary**

- Multiple cervical artery dissections may indicate fibromuscular dysplasia, warranting comprehensive evaluation and treatment.

## 1 Introduction

Fibromuscular dysplasia (FMD) is a vascular disorder affecting primarily medium‐sized arteries, although it can involve arteries of any size and territory [1]. It is a multifactorial angiopathy not associated with atherosclerosis or inflammatory diseases, more commonly affecting young women and smokers [2]. Diagnosis of FMD requires at least one focal or multifocal arterial lesion, with involvement commonly observed in cervical and renal arteries [2]. Cerebrovascular involvement in FMD is considered as prevalent as renal involvement, although fewer studies exist in the general population [1]. Imaging plays a crucial role in diagnosis, with CTA, MRA, and DSA being key modalities. Each has specific advantages: CTA offers rapid assessment, MRA provides vessel wall imaging, and DSA remains the gold standard for confirming FMD characteristics. Here, we present a case of right internal carotid artery (ICA) and bilateral vertebral arteries (VAs) dissection associated with FMD.

## 2 Case History

A 30‐year‐old female presented with progressive holocranial headache over 2 weeks, accompanied by neck pain, vertigo, and vomiting triggered by physical exertion at the gym. She had a history of migraine, alcoholism, deep venous thrombosis post‐plastic surgery, and use of steroids (oxandrolone, stanozolol) and oral contraceptives. Neurological examination revealed ataxic gait and bilateral dysmetria.

### 2.1 Examination/Presentation

A head CT scan showed frontal subarachnoid hemorrhage (SAH) and hypodensities in the parieto‐occipital and cerebellar hemispheres, with cerebellar swelling compressing the fourth ventricle and slight ventricular dilation. CT angiography indicated irregular arterial walls and left vertebral artery (VA) occlusion, suggestive of thrombosis. Rapid neurological deterioration led to orotracheal intubation, external ventricular drainage (EVD), and posterior fossa craniectomy.

### 2.2 Methods (Differential Diagnosis, Investigations, and Treatment)

**FIGURE 1:**
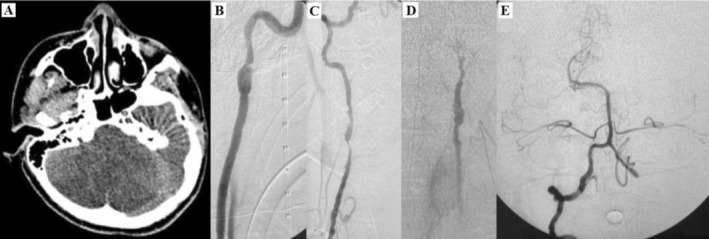
Head CT demonstrates diffuse hypoattenuation of cerebellar hemispheres (A). CTA identified luminal irregularities, prompting further investigation. Angiography reveals dissections in the right internal carotid artery at the craniocervical transition (B), V2 and V3 segments of the right vertebral artery (C), dysplastic V2 segment of the left vertebral artery with distal occlusion (D), and intracranial occlusions in the right posterior inferior cerebellar artery and V4 segment of the left vertebral artery (E). These findings are consistent with multifocal fibromuscular dysplasia.

Following multidisciplinary consultation, the patient was started on 100 mg acetylsalicylic acid and 40 mg simvastatin daily. To further investigate etiology, we employed a multimodal imaging strategy:

Postoperative digital arteriography (DA) revealed (Figure 1):

- CTA and MRA identified arterial dissections and luminal irregularities, prompting further assessment.
- DSA was performed to confirm FMD by detecting the characteristic “string of beads” appearance.
- Dissecting aneurysm in the right internal carotid artery (ICA) with 80% stenosis.
- Irregularities and aneurysm in the cavernous segment of the left ICA.
- Wall irregularities and dissection causing stenosis in the cervical segment of the right VA.
- Dissecting aneurysm and occlusion in the left VA.

### 2.3 Results (Outcome and Follow‐Up)

The patient remained sedated for 3 days, extubated on the fifth day, and had the EVD removed on the seventh day without signs of hydrocephalus. Upon examination, she exhibited dysphonia, dysarthria, ophthalmoplegia on upward gaze, dysmetria, dysdiadochokinesia, and global hyperreflexia. After 20 days of hospitalization, she was transferred to a rehabilitation facility.

In 2 months of outpatient follow‐up, the patient reported significant improvement with minimal deficits: slight dysmetria, bilateral dysdiadochokinesia, and positional vertigo, able to perform activities of daily living independently.

## 3 Discussion

Cervical artery dissections (CAD) can be classified as traumatic or spontaneous, with the latter often occurring after minor trauma or physical exertion [3]. Spontaneous CAD is a major cause of ischemic stroke in young adults, accounting for approximately 20% of strokes in individuals under 45 years old [3, 4]. The pathophysiology involves intramural hematoma forming within the arterial wall, leading to luminal stenosis, occlusion, or pseudoaneurysm formation. The most affected arteries are the internal carotid arteries (ICAs) and vertebral arteries (VAs), and patients often present with headache, neck pain, or focal neurological deficits [4].

One of the most crucial predisposing factors for CAD is an underlying arteriopathy, such as fibromuscular dysplasia (FMD). FMD is a noninflammatory, nonatherosclerotic vascular disorder affecting medium‐sized arteries, with an estimated prevalence of 5.6%–21% in patients with CAD, increasing to nearly 30% in those with multivessel dissections [3, 4, 5, 6]. The strong association between FMD and CAD highlights the need for systematic vascular screening in patients with multiple dissections, particularly young women [5, 6].

Our case is unique due to the simultaneous involvement of three cervical arteries (right ICA and both VAs), a pattern rarely documented in the literature. Although bilateral cervical artery dissections are reported in 8%–14% of cases, three‐vessel dissections remain uncommon (6.5%) [5]. The specific combination of one ICA and two VAs is exceedingly rare, reinforcing the necessity of considering FMD as an underlying etiology in such cases. Interestingly, most FMD‐associated CADs involve the middle to distal ICA (95%) and the V3 segment of the VA, whereas our patient had dissections in the proximal ICA and V2 segments of the VAs, suggesting an atypical distribution [7, 8].

Another significant aspect of this case is the presence of subarachnoid hemorrhage (SAH), a complication seen in 5%–11% of spontaneous CADs, usually due to dissecting aneurysm rupture [8]. The management of SAH in patients with FMD‐related dissections follows standard protocols, including vigilant blood pressure control and, when necessary, endovascular or surgical intervention [8]. Our patient underwent posterior fossa decompressive craniectomy and external ventricular drainage, which were critical in preventing brainstem compression and hydrocephalus.

Risk factors in this case are also warranting discussion [9]. The patient had a history of migraine, oral contraceptive use, and anabolic steroid consumption, all of which have been linked to CAD [10, 11, 12, 13]. Migraine is found in 18%–23% of CAD patients and is often associated with a hyperreactive vascular response [10]. Anabolic steroids have been implicated in cases of vertebral artery dissection, possibly due to their effects on vascular integrity and endothelial function [13, 14, 15, 16]. Additionally, oral contraceptives may contribute to a hypercoagulable state, increasing the risk of vascular injury [5]. The interplay of these factors may have contributed to arterial fragility in our patient, further emphasizing the multifactorial nature of CAD in young adults.

Imaging is paramount in diagnosing CAD and underlying FMD. CTA is a rapid and widely available tool for initial assessment, capable of detecting luminal irregularities, stenosis, or aneurysms. MRA provides superior vessel wall imaging, aiding in differentiating intramural hematomas from atherosclerotic lesions. DSA remains the gold standard, allowing for the identification of the characteristic “string of beads” pattern in FMD [3, 7]. In our case, the integration of these modalities was essential in confirming the diagnosis.

Management strategies for FMD‐associated dissections focus on antithrombotic therapy, symptom control, and risk factor modification. In cases of ischemic stroke without hemorrhagic transformation, thrombolysis and thrombectomy may be considered, though caution is required due to vessel fragility [2, 7]. Angioplasty or stenting is reserved for cases of symptomatic stenosis, recurrent ischemia, or refractory symptoms [2]. Our patient demonstrated significant improvement after 2 months of dual antiplatelet therapy and rehabilitation, highlighting the potential for favorable outcomes despite the severity of the initial presentation.

This case underscores the importance of early recognition, multimodal imaging, and individualized management in FMD‐related CADs. Furthermore, it contributes to the growing evidence that multivessel dissections should prompt an aggressive search for an underlying arteriopathy, particularly in young women presenting with ischemic stroke or SAH.

## 4 Conclusion

FMD remains an underdiagnosed yet significant contributor to spontaneous CADs. This case report highlights the critical importance of maintaining a high index of suspicion for FMD in patients presenting with multiple cervical artery dissections, particularly when the pattern of involvement is atypical. While most FMD‐associated dissections affect the middle to distal ICA and V3 segment of the VA, the involvement of the proximal ICA and V2 segments in our case suggests a broader spectrum of arterial vulnerability in FMD patients. Given the potential for life‐threatening complications such as subarachnoid hemorrhage, ischemic stroke, and hydrocephalus, prompt diagnosis using multimodal imaging is essential. CTA, MRA, and DSA should be utilized in a complementary manner to ensure accurate detection of dissections and underlying arteriopathies. Management strategies must be tailored to the individual patient, considering factors such as the extent of arterial involvement, presence of ischemic or hemorrhagic complications, and risk of recurrence. Our case demonstrates that even extensive multivessel dissections can result in good functional outcomes with appropriate intervention and rehabilitation.

## Author Contributions

**Julia Goginski:** conceptualization, resources, writing – original draft. **Gianluca Scalia:** supervision, validation, visualization, writing – review and editing. **Renato Fedatto Beraldo:** conceptualization, data curation, writing – original draft. **Ricardo Nascimento Brito:** data curation, formal analysis. **Lohana Pompelli Scapatici:** resources, supervision. **Bruno Liebl:** resources, validation. **Zeferino Demartini Junior:** formal analysis, visualization, writing – original draft. **Giovanni Federico Nicoletti:** supervision, visualization. **Bipin Chaurasia:** supervision, visualization.

## Disclosure

The authors have nothing to report.

## Ethics Statement

This study was performed in accordance with the ethical standards and the World Medical Association Declaration of Helsinki and ICMJE Recommendations. It was previously approved by the Research Ethical Committee of PUCPR under the number 6.548.232, and all patient identification was removed to preserve anonymity.

## Consent

A full and detailed consent from the patient has been taken. The patient's identity has been adequately anonymized. The authors certify that they have obtained all appropriate patient consent forms. In the form, the patient has given his‐her‐their consent for his‐her‐their images and other clinical information to be reported in the journal. The patients understand that their names and initials will not be published and due efforts will be made to conceal their identity.

## Conflicts of Interest

The authors declare no conflicts of interest.

## Data Availability

The authors have nothing to report.
